# [^99cm^Tc]Tc-PSMA-I&S-SPECT/CT: experience in prostate cancer imaging in an outpatient center

**DOI:** 10.1186/s13550-020-00635-z

**Published:** 2020-05-07

**Authors:** P. Werner, C. Neumann, M. Eiber, H. J. Wester, M. Schottelius

**Affiliations:** 1 Nuclear Medicine Neumann, Outpatient Practice for Nuclear Medicine, Leipzig, Germany; 2 Department of Nuclear Medicine, Technical University of Munich, Klinikum rechts der Isar, Munich, Germany; 3 Pharmaceutical Radiochemistry, Technical University of Munich, Klinikum rechts der Isar, Munich, Germany; 4 Department of Nuclear Medicine, Centre Hospitalier Universitaire Vaudois, and Department of Oncology, University of Lausanne, Lausanne, Switzerland

**Keywords:** PSMA, SPECT, [^99m^Tc]Tc-PSMA-I&S, Scintigraphy, Prostate cancer

## Abstract

**Background:**

Prostate-specific membrane antigen (PSMA) SPECT imaging in prostate cancer (PCa) could be a valuable alternative in regions where access to PSMA-PET imaging is restricted. [^99m^Tc]Tc-PSMA-I&S is a new ^99m^Tc-labeled PSMA-targeting SPECT agent, initially developed for radio-guided surgery. We report on the diagnostic use of [^99m^Tc]Tc-PSMA-I&S-SPECT/CT in PCa.

**Results:**

[^99m^Tc]Tc-PSMA-I&S-SPECT/CT was performed and evaluated in 210 outpatients with PCa at a single center. Patients were imaged for biochemical recurrence (BCR, *n* = 152, mean PSA 8.7 ng/ml), for primary staging of high-risk PCa (*n* = 12, mean PSA 393 ng/ml), and restaging in advanced recurrent PCa (*n* = 46, mean PSA 101.3 ng/ml). Number and location of positive lesions were determined for the different subgroups. For BCR, detection rates were calculated, defined as the proportion of scans with at least one PSMA-positive lesion.

PSMA positive lesions were detected in 65.2% of all 210 patients. Tumor tissue was mainly detected in lymph nodes (59%), in the bone (42%), and in the prostate (fossa) (28%). In the subgroup of patients referred for detection of BCR the detection rate increased from 20% at a PSA level < 1 ng/ml to 82.9% and 100% at PSA levels > 4 ng/ml and > 10 ng/ml, respectively. In the subgroup of high-risk patients referred for primary staging, 42% demonstrated metastatic disease. Restaging of advanced recurrent PCa revealed detectability of PSMA positive tumor lesions in 85% of the scans.

**Conclusions:**

[^99m^Tc]Tc-PSMA-I&S-SPECT/CT was useful in PSMA-targeted imaging of PCa at various clinical stages. At low PSA levels (< 4 ng/ml), detection rates of [^99m^Tc]Tc-PSMA-I&S-SPECT/CT in BCR are clearly inferior to data reported for PET-imaging and should thus only be considered for lesion detection if imaging with PET is unavailable. However, at higher PSA levels (> 4 ng/ml) [^99m^Tc]Tc-PSMA-I&S-SPECT/CT provides high detection rates in BCR. [^99m^Tc]Tc-PSMA-I&S-SPECT/CT can also be used for primary staging and for restaging of advanced recurrent PCa. However, further studies are needed to assess the clinical value in these indications.

## Background

During the last decade, prostate-specific membrane antigen (PSMA)-targeted positron emission tomography (PET) has become a substantial part in imaging of prostate cancer (PCa). In some national guidelines, it is the preferred method for lesion detection in biochemical relapse (BCR) after primary treatment and is mandatory prior to PSMA-directed radionuclide therapy [1]. Although the exact role of PSMA-PET for numerous other indications (e.g., primary staging in high-risk cancer, targeted radiation therapy) is still under investigation, overall clinical demand, and examination numbers are rising [2].

In many countries, however, access to PET is often restricted and overall instrumentation and radionuclide costs are higher as compared to single photon emission tomography (SPECT) [3]. Therefore, the potential of SPECT tracers in the management of PCa is increasingly explored [4–6]

In recent studies, it was demonstrated that the PSMA inhibitor and SPECT-compatible agent [^99m^Tc]Tc-MIP-1404 detected PSMA-positive lesions with high sensitivity in patients with biochemical recurrence of PCa (70% and 77% of examined patients, respectively) [4, 7]. The same group reported on the use of [^99m^Tc]Tc-MIP-1404-SPECT/CT for whole-body primary staging in 95 treatment-naïve PCa patients [8]. They reported a high accuracy and a low inter-observer variability for diagnosis of PCa (90 of 93 patients) and for the detection of lymph node metastases, bone metastases and disseminated bone metastases in 16, 9, and 3 patients, respectively.

The use of another ^99m^Tc labeled PSMA ligand ([^99m^Tc]Tc-HYNIC-Glu-Urea-A) in 39 patients with biochemical relapse of PCa yielded higher diagnostic efficiencies of PSMA-SPECT/CT (computed tomography) than bone scan and magnetic resonance imaging (MRI; metastatic lesions in 78%, 34%, 42% of the patients, respectively) [6]. The same study also demonstrated a change of the therapeutic strategy with the aid of [^99m^Tc]Tc-PSMA-SPECT/CT in 79% of the patients.

Another PSMA-targeted compound, [^99m^Tc]Tc-PSMA-I&S (for imaging and surgery), was introduced in 2016 by Robu et al. [9]. It was initially developed for radio-guided surgery and is characterized by slow whole-body clearance and thus prolonged availability for internalization into the tumor cells. This leads to gradually increasing lesion-to-background ratios up to 21 h after injection, making it suitable for intraoperative detection and resection of PSMA-positive lymph node metastases [9]. Due to the ease of tracer preparation via a robust and reliable labeling procedure, [^99m^Tc]Tc-PSMA-I&S can potentially be used in the outpatient setting for SPECT imaging [9]. However, until now, there is scarce evidence on the value of [^99m^Tc]Tc-PSMA-I&S as a diagnostic SPECT tracer. Our aim was to assess the diagnostic use of [^99m^Tc]Tc-PSMA-I&S-SPECT/CT in a large number of patients with different stages of PCa.

## Materials and methods

### Patients

This is a retrospective analysis of 210 outpatients with histological proven PCa. All patients were referred by urologists, oncologists, radiotherapist, and nuclear medicine physicians to an outpatient nuclear medicine praxis. They underwent [^99m^Tc]Tc-PSMA-I&S-SPECT/CT between June 2016 and December 2018 on a compassionate use basis according to German Medicinal Products Act (AMG §13.2b) and the responsible regulatory bodies (Government of Oberbayern and Saxony, Germany). Written consent was obtained from all patients. The median prostate-specific antigen (PSA) value at the time of imaging was 50.0 ng/ml (range 0.01 to 3500 ng/ml). Table 1 gives an overview about the indications and PSA-levels of all patients included in the retrospective analysis. For data analysis, all information provided by referring physicians at the time of referral was used.

**Table 1:** Patient’s characteristics and indications for [^99m^Tc]Tc-PSMA-I&S-SPECT/CT

	***N***	Age [years]	Mean PSA [ng/ml]
Total	210	73 (± 8.5)	50 (± 270)
Indications			
biochemical recurrence (BCR)	152 (72.4%)	72.8 (± 8.8)	8.7 (± 32)
primary staging in high-risk PCa	12 (5.7%)	72.7 (± 8.6)	393 (± 982)
restaging in advanced recurrent PCa (antiandrogen therapy, chemotherapy, radiation therapy, ^177^Lu-PSMA therapy)	46 (21.9%)	74.2 (± 8.1)	101.3 (± 242)

### Procedure and imaging

[^99m^Tc]Tc-PSMA-I&S was prepared as previously described [9]. A mean activity of 761.5 (± 49) MBq was administered as an intravenous bolus. After tracer injection in the morning hours, the patients left the outpatient imaging center and returned 5 h later. All patients underwent whole body planar imaging and whole body [^99m^Tc]Tc-PSMA-I&S-SPECT with non-contrast-enhanced (low-dose) CT. The low-dose CT was used for attenuation correction and anatomic localization.

SPECT imaging was performed 5 h post injection (p.i.) on an Optima NM/CT 640 SPECT/CT system (GE Healthcare). Planar whole-body imaging (LEHR collimation, pixel size 2.4 mm, scan speed 19 cm/min) was followed by 2 SPECT bed positions covering the body trunk (LEHR collimation, 128 × 128 matrix, 120 projections over 360°, 60 stops at 2 projections, dwell time of 16 s per stop). The low-dose CT was acquired with 120 kV and 30 mA with a matrix size of 512 × 512.

**Fig. 1:**
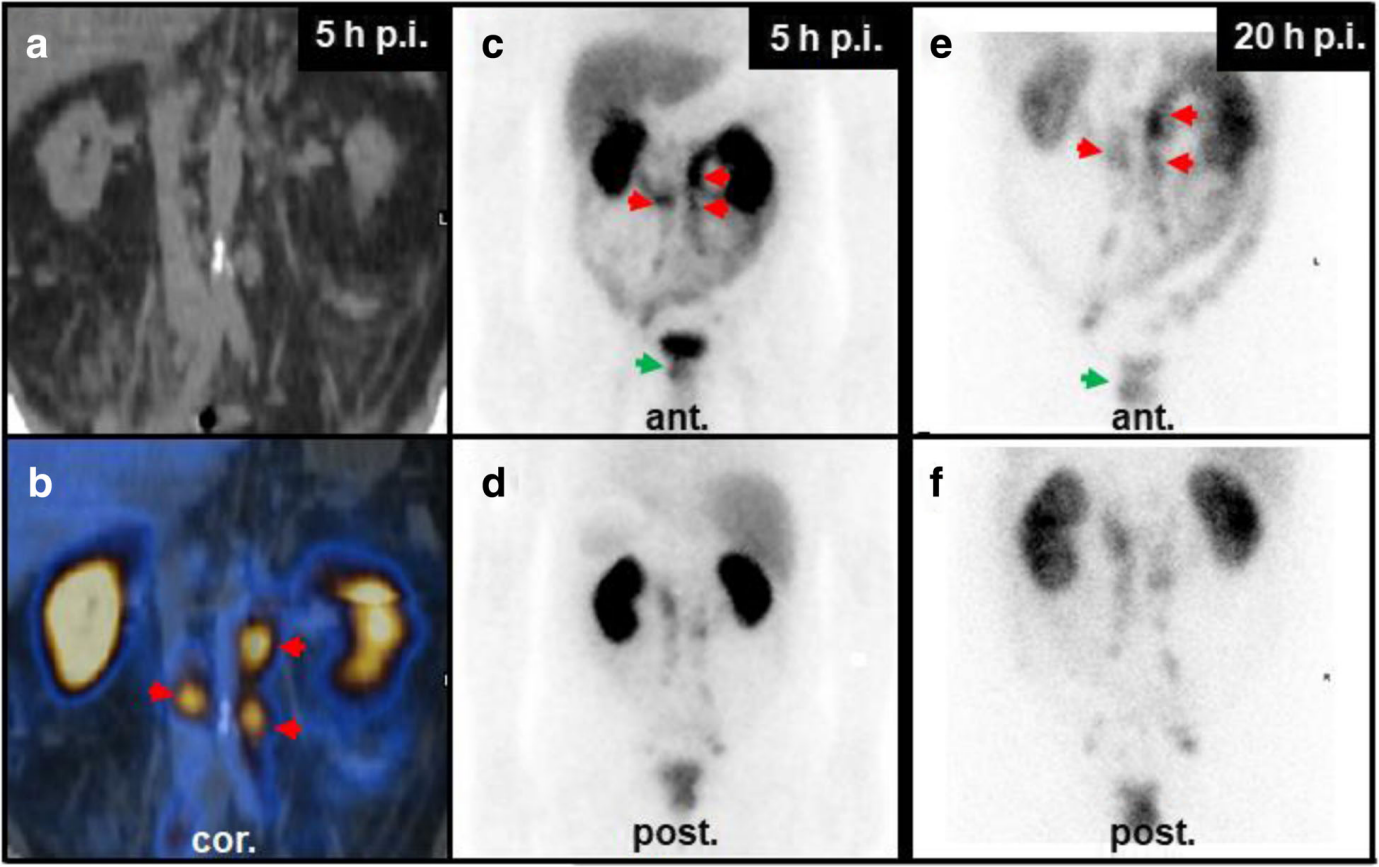
SPECT/CT at 5 h post injection (p.i.) and pairs of ventral/dorsal planar images at 5 h and 20 h p.i. of [^99m^Tc]Tc-PSMA-I&S. **a**, **b** SPECT/CT suggested paraaortic lymph node metastases, but the planar scintigraphy (**c**, **d**) shows significant hepatobillary and gastrointestinal uptake and suggests urinary secretion. To further distinguish between tumor uptake in paraaortic lymph nodes and unspecific uptake in the ureter (red arrows), a late planar image was taken. **e**, **f** Planar scintigraphy at 20 h p.i. Paraaortic uptake remained high and was thus classified as lymph node metastases (red arrows). Note: there was a tracer accumulation in the primary tumor over time, resulting in a better contrast between unspecific bladder signal and the [^99m^Tc]Tc-PSMA-I&S uptake in the prostate (green arrows). Late imaging was restricted to a single bed planar scintigraphy because the low activity at 20 h p.i. would have led to inappropriately high examination times (~ 60 min for planar whole body scintigraphy and whole body SPECT/CT)

Images were analyzed by visual inspection, evaluated, and discussed by two experienced readers. Physiological uptake in the gall bladder, kidneys, salivary and lacrimal glands, spleen, liver, thyroid, and digestive and urinary tract was ignored. Focal uptake that could not be explained by gastrointestinal uptake or urinary secretion was interpreted suspicious of prostate cancer (PSMA-positive) [6]. Findings were classified by two experienced board certified nuclear medicine physicians in consensus. In cases where during primary reading at the end of the scan indeterminate results (e.g., tumor uptake vs. tracer excretion in the urinary tract) were present, additional imaging at the discretion of the responsible physician was ordered. In two scans, the readers could not agree on either tumorous uptake or unspecific tracer excretion, and late imaging was thus performed. In one case, planar imaging was sufficient (one 40 cm bed position, 256 matrix, stop after 1200 s or 300 kcts, see Fig. 1), and in the other case, a late SPECT/CT was performed to confirm unspecific gastrointestinal uptake (SPECT dwell time of 40 s instead of 16 s per stop).

### Statistical analyses

Number and location of PSMA-positive lesions in [^99m^Tc]Tc-PSMA-I&S-SPECT are reported. All scans with at least one suspicious finding in lymph nodes, in the bone, in the prostate, the prostatic fossa (after prior resection of the prostate), the lung, the liver, the peritoneum or other soft tissue were classified as “positive” scans. Sensitivity and specificity could not be calculated, because information on histopathology outcomes was not available. Detection rates were calculated for the subgroup of BCR [6], whereas detection rates were defined as the number of “positive” scans divided by the total number of scans and was presented as a percentage with 95% confidence Interval (CI). Statistical analyses were performed with IBM SPSS Statistics ver. 24.0 (IBM Co., Armonk, NY, USA).

## Results

### Biodistribution

Due to urinary and hepatobiliary clearance of [^99m^Tc]Tc-PSMA-I&S, a significant degree of hepatic, gastrointestinal and urinary tracer uptake was still observed at 5 h p.i. (Fig. 1a–f). However, low-dose CT generally allowed for good discrimination between unspecific uptake and tumor lesions. Only in two cases, a late scan (20 h p.i.) was performed to improve distinction between urinary or gastrointestinal excretion and tumor tissue. At 20 h p.i., higher contrast between lesions and background was observed, as it was shown previously [9], which was achieved at the cost of a longer imaging time.

### Number and location of tumor lesions

**Fig. 2:**
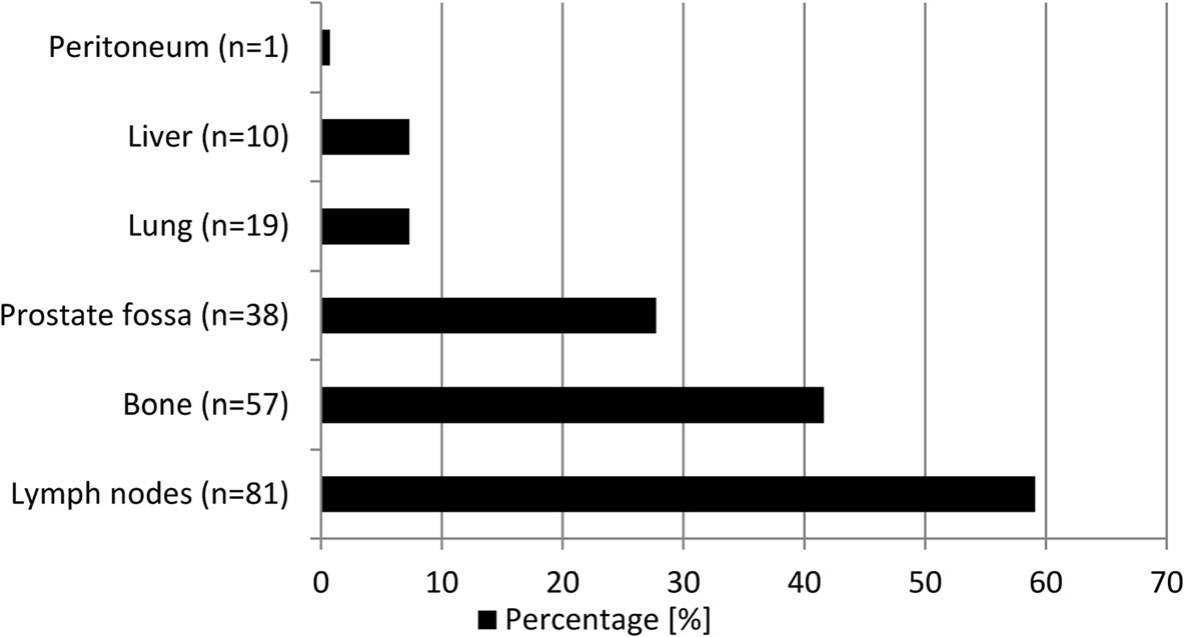
Location of tumor tissue in 137 PSMA-positive scans

In 137 (65.2%) of 210 [^99m^Tc]Tc-PSMA-I&S-SPECT/CT scans at least one PSMA-positive lesion was detected. In the positive scans, tumor-related tracer accumulation was detected in lymph nodes (59.1%), in the bone (41.6%), in the prostate (if still present), or in the prostate fossa (27.7%) (Fig. 2).

#### Biochemical recurrence (BCR)

**Fig. 3:**
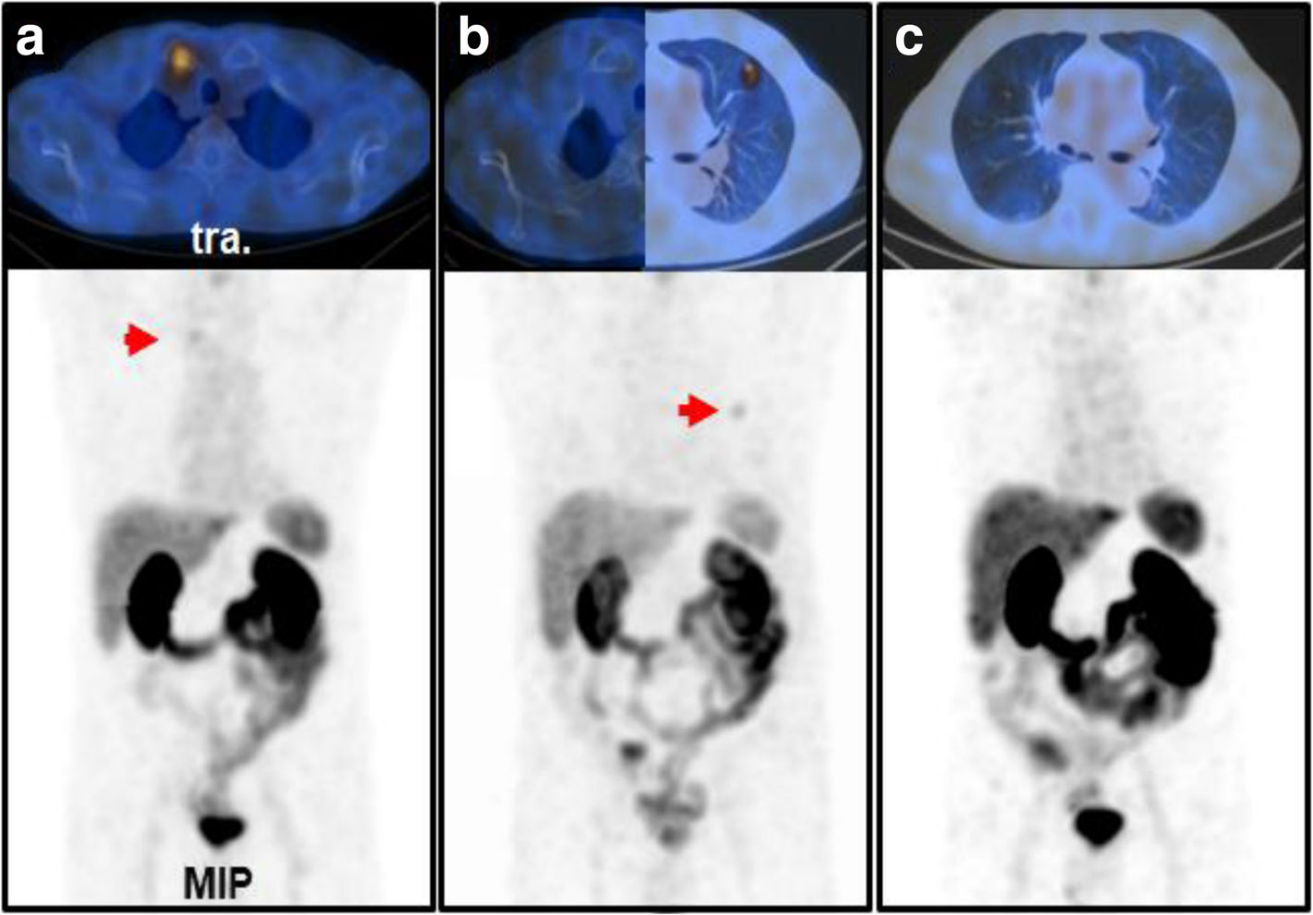
Lesion detection in BCR and monitoring of radiation therapy in a 72-year-old patient with Gleason 7 PCa, who underwent primary surgery and salvage radiation therapy. **a** Cancer recurrence was detected in the right clavicle (PSA 3.8 ng/ml) which was followed by targeted radiation. **b** 6 months later the clavicle was unremarkable but another recurrence was detected in the lung (PSA 6.8 ng/ml) again followed by precision radiotherapy. **c** Another 6 months later, no tumor recurrence was detected (PSA 0.56 ng/ml). Note: the apparent focal PSMA-positivity in the liver (**c**) could not be confirmed in the cross-sectional planes and was not categorized as PSMA-positive

PSMA-positive lesions were detected in 57.6% (95% confidence interval (CI) 0.50–0.65) of patients with BCR. Detection rate was 20% (95% CI, 0.10–0.34) at PSA levels 0 to 1 ng/ml (average PSA level of 0.44 ng/ml) and increased to 55.2% (95% CI, 0.42–0.68) at an average PSA of 2.05 ng/ml. Detection rate on patient level exceeded 80% in patients with PSA levels ≥ 4 (≥ 4: 82.9% (95% CI, 0.68 to − 0.93), > 10: 100%). Details on the detection efficacy are presented in Table 2. Patient examples are shown in Fig. 3.

**Table 2:** Lesion detection by [^99m^Tc]Tc-PSMA-I&S-SPECT/CT in BCR

PSA range [ng/ml]	Patients	Ø PSA [ng/ml]	Detection rate [%]
0–1	*n* = 41	0.44 ± 0.29	20 (95% CI, 0.1–0.34)
> 1–4	*n* = 58	2.05 ± 0.89	55.2 (95% CI, 0.42–0.68)
> 4–10	*n* = 35	4.74 ± 2.02	82.9 (95% CI, 0.68–0.93)
> 10	*n* = 18	52.3 ± 81.8	100

#### Primary staging

**Fig. 4:**
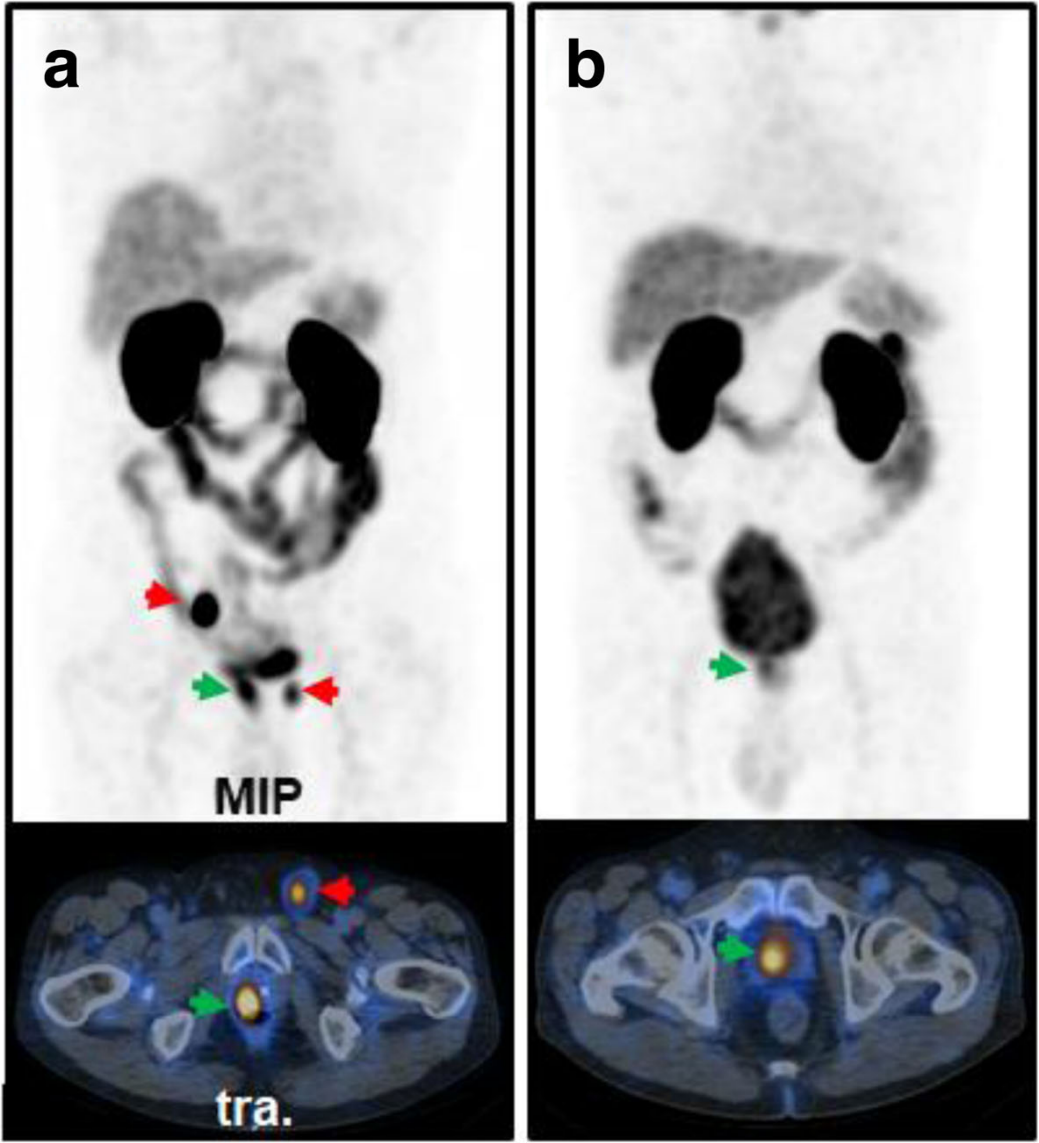
[^99m^Tc]Tc-PSMA-I&S-SPECT/CT in primary high risk PCa. **a** 71-year-old man with Gleason 9 PCa (PSA 46 ng/ml). Apart from the primary tumor (green arrow), increased PSMA expression was detected in one lymph node in the right iliac region and in the left inguinal region (red arrows)(TxN1M0). **b** 77-year-old man with Gleason 9 PCa (PSA 80 ng/ml). PSMA expression was increased in the primary tumor only (green arrow), no metastases were detected (TxN0M0)

In addition, 12 patients (mean PSA 393.9 ng/ml ± 903) were referred for [^99m^Tc]Tc-PSMA-I&S-SPECT/CT for primary staging of high-risk PCa (classified by D’ Ámico criteria). In 11 out of 12 patients, the primary tumor demonstrated high tracer accumulation. In 4 patients, lymph node metastases were detected, in 4 patients bone metastases were detected and in 1 patient a pulmonary metastasis was detected. This resulted in an upstaging after [^99m^Tc]Tc-PSMA-I&S-SPECT/CT in five patients from an initial N0M0 disease to TxN1M0, TxN1M1b, TxN0M1b, TxN1M1c, and TxN1M1a,b, respectively. Patient examples are presented in Fig. 4. In seven patients, no regional or distant metastases were present (TxN0M0).

#### Restaging in advanced recurrent PCa

**Fig. 5:**
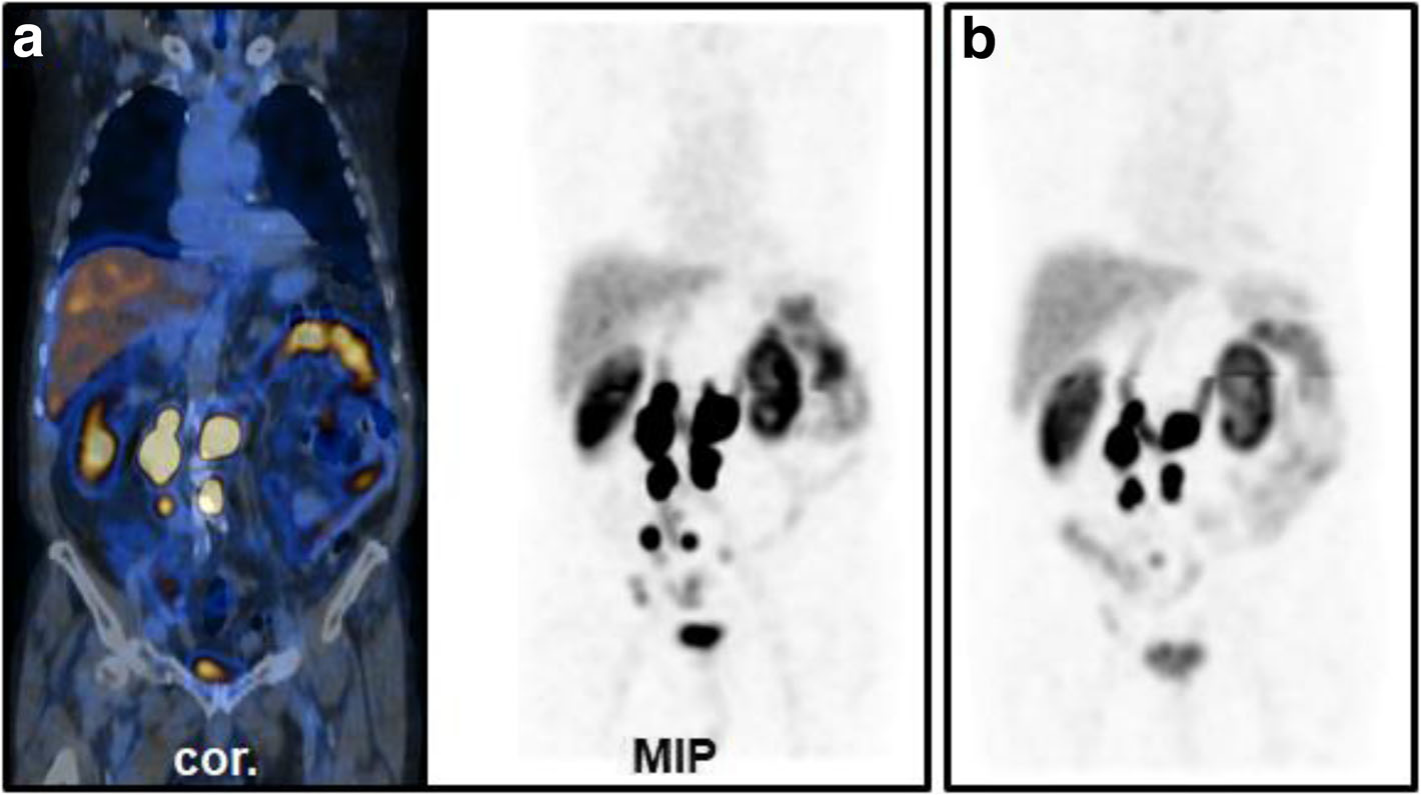
[^99m^Tc]Tc-PSMA-I&S-SPECT/CT of an 82-year-old patient with mCRPC and disease progression under chemotherapy (docetaxel). **a** Pretherapeutic tumor spread (PSA 137 ng/ml). **b** Restaging 3 months after the second cycle of ^177^Lu-PSMA therapy (PSA 60 ng/ml)

**Fig. 6:**
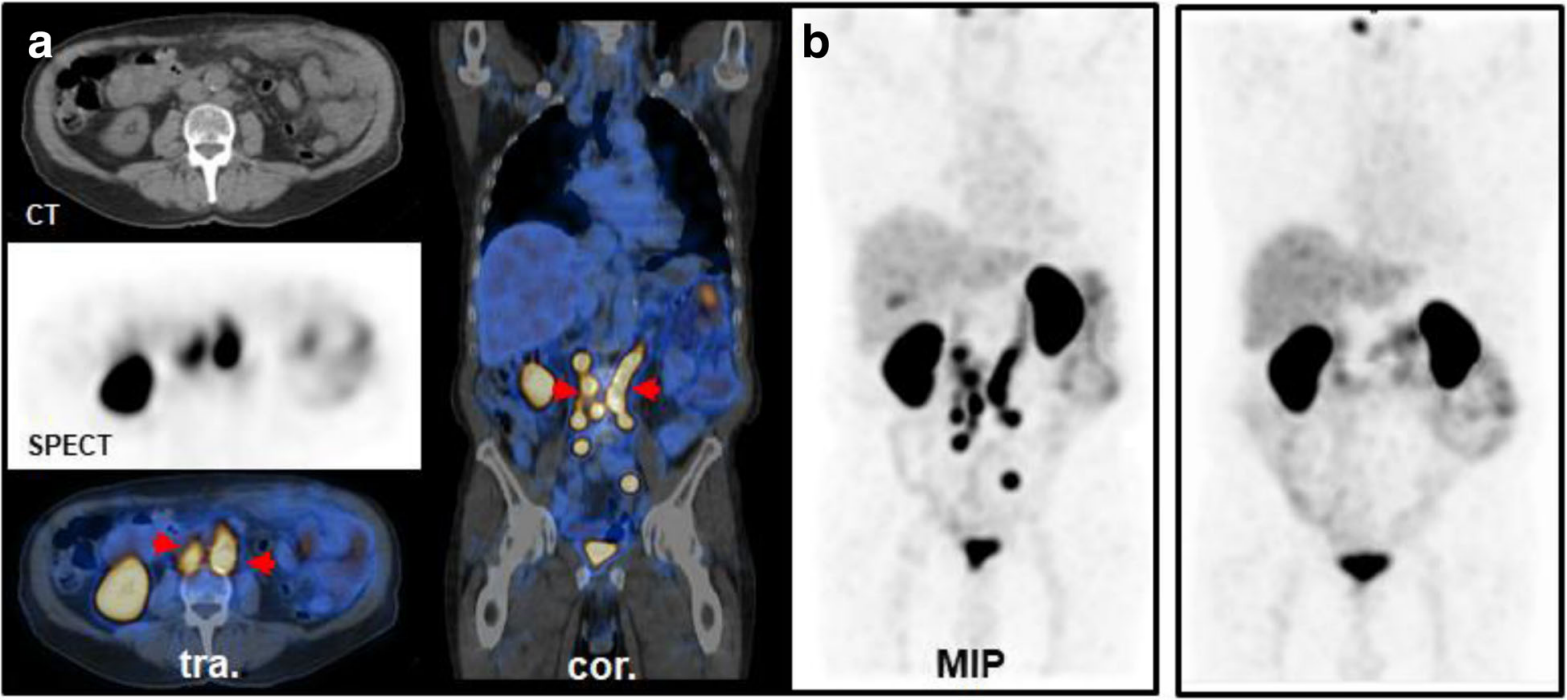
[^99m^Tc]Tc-PSMA-I&S-SPECT/CT of a 71-year-old patient with mCRPC and disease progression and severe fatigue after the second cycle of chemotherapy (docetaxel). **a** Pretherapeutic tumor spread in paraaortic and pelvic lymph nodes (PSA 335 ng/ml). **b** Restaging 3 months after 2 cycles of ^177^Lu-PSMA therapy (PSA 0.6 ng/ml). [^99m^Tc]Tc-PSMA-I&S-SPECT/CT was negative

**Fig. 7:**
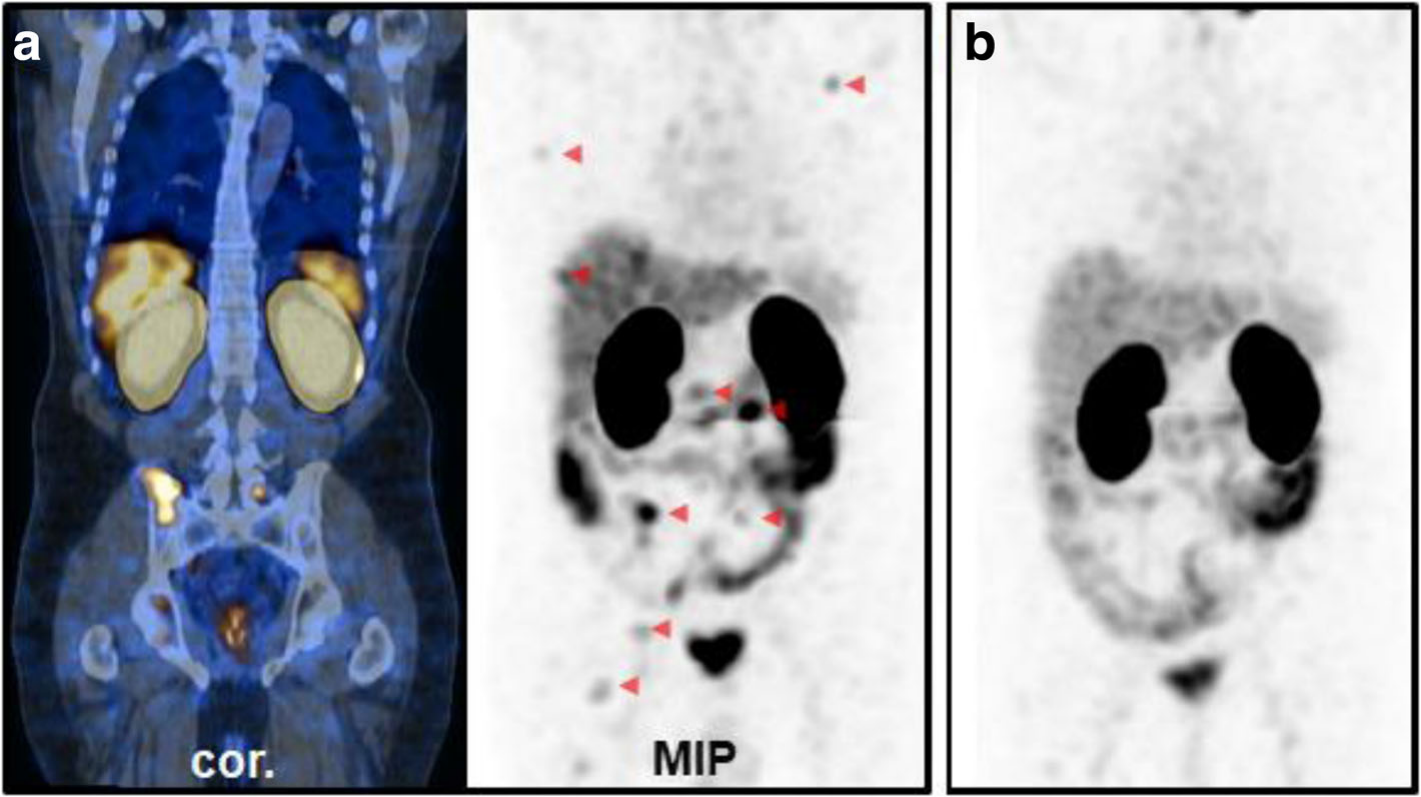
[^99m^Tc]Tc-PSMA-I&S-SPECT/CT of a 59-year-old patient with radical prostatovesiculectomy and salvage radiotherapy. According to morphological imaging 2 years later osseous metastases were suspected. **a** At baseline, multiple osseous and lymph node metastases (partly marked with red arrows) were detected with mostly mild PSMA expression (PSA 4.75 ng/ml). **b** Follow-up scan 4 months later under antihormonal therapy (PSA 0.01 ng/ml). [^99m^Tc]Tc-PSMA-I&S-SPECT/CT was negative

In advanced recurrent PCa, [^99m^Tc]Tc-PSMA-I&S scans (*n* = 46; mean PSA 101.3 ng/ml ± 242) were requested for monitoring of antihormonal therapy (*n* = 18), ^177^Lu-PSMA therapy (*n* = 13), chemotherapy (*n* = 8), and radiation therapy (*n* = 7). PSMA-positive tumor tissue was detected in 39 scans (84.8%). Metastatic disease of the lymph nodes, bone, liver and the lung was present in 27, 25, 8, and 3 patients, respectively. Local recurrence was present in 9 patients. In 7 patients, baseline scans and follow scans up after therapy were available (Figs. 3b, c, 5, 6, and 7). Of those, favorable therapy response could be confirmed in all four patients that underwent ^177^Lu-PSMA therapy, in one patient that underwent targeted radiation therapy and in one patient with antihormonal treatment. In one patient, the follow-up scan showed progressive disease under chemotherapy.

## Discussion

In this retrospective single-center study, [^99m^Tc]Tc-PSMA-I&S-SPECT/CT was used for tumor detection in PCa, because timely PSMA-PET was not available. Indications were lesion detection in BCR, primary staging, general restaging, and therapy monitoring. For these indications, [^99m^Tc]Tc-PSMA-I&S was used and found to be a reliable and suitable tracer for PSMA-targeted SPECT imaging. The imaging procedure performed within 5 h post injection could be well integrated into the work schedule of an outpatient nuclear medicine practice. Only in two cases imaging at a later time point (20 h post injection) was needed.

### Detection rate in BCR

**Fig. 8:**
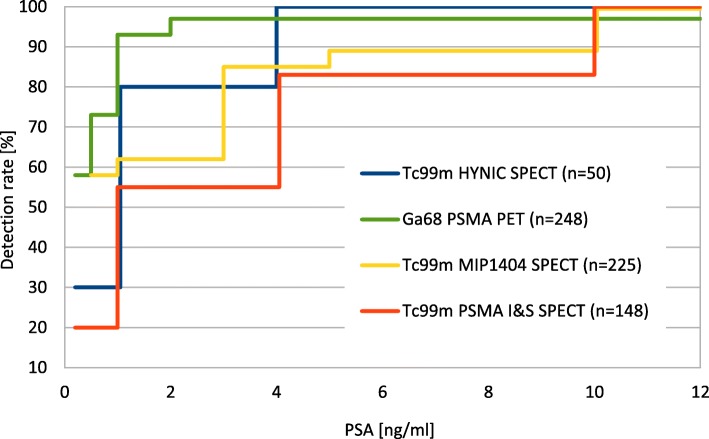
Detection rates of different PSMA ligands in recurrent PCa. The figure was adapted from PSMA imaging detection rates from Su H-C. et al. [6] (Blue), Eiber M. et al. [10] (green), Schmidkonz C. et al. [4] (yellow), and the present study (red)

The overall detection rate was 20% and 55% at PSA levels lower than 1 and 4 ng/ml, respectively. At PSA levels greater than or equal 4 ng/ml, the detection rate was 83%, reaching 100% at PSA levels ≥ 10 ng/ml (Fig. 8). Recent studies with another dedicated PSMA-targeted SPECT agent ([^99m^Tc]Tc-MIP-1404) reported higher detection rates in patients with recurrent of PCa [4, 7]. In those studies, a detection rate of 90% and 91.4% was observed at PSA levels ≥ 2 ng/ml. Another group also reported higher detection rates for SPECT in recurrent PCa [6]. Here, a detection rate of 80% and 100% at PSA levels ≥ 1 and 4 ng/ml, respectively, was achieved. Figure 8 provides an overview on PSMA-SPECT detection rates in comparison to PSMA-PET.

Upon careful comparison with the study of Schmidkonz et al. [4], it becomes obvious that the difference between [^99m^Tc]Tc-MIP-1404 and [^99m^Tc]Tc-PSMA-I&S is more pronounced at very low PSA levels. Schmidkonz et al. report a detection rate of 58% at an average PSA level of 0.47 ng/ml, while in this study a detection rate of 20% was observed at the same PSA level (ØPSA 0.44 ng/l). A lower difference is present at higher PSA levels. At an average PSA level of 1.9 ng/ml, Schmidkonz et al. reported a detection rate of 62%, whereas we found a detection rate of 55% at an average PSA level of 2.1 ng/ml.

The somewhat lower detection rate observed in this analysis can be related to several reasons. Firstly, the tracer kinetics lead to a relatively high gastrointestinal tracer uptake besides its urinary excretion. Secondly, the results in this study are exclusively based on visual image inspection and interpretation, and quantitative analysis was not performed.

### Primary staging

In our study, the primary tumor demonstrated pathological tracer accumulation in 11 of 12 patients. This is in line with earlier studies from Goffin et al. [11] and Schmidkonz et al. [8]. They reported on the use of [^99m^Tc]Tc-MIP-1404-SPECT/CT prior to surgery in 104 and 93 patients and found a positivity rate of the primary tumor of 94% and 97%, respectively.

But the goal of PSMA imaging in the primary diagnosis is not to characterize the primary tumor, but to detect the initial metastatic burden [10]. Schmidkonz et al. observed lymph node metastases in 17.2% and bone metastasis in 12.9% of the cases. Goffin et al. showed a low sensitivity (50%), but good specificity (87%) in detecting histologically proven lymph node involvement in 32% of the patients. In our study lymph node metastases were detected in 4 patients, bone metastases were detected in 4 patients and a pulmonary metastasis was detected in 1 patient.

The number of patients referred for primary staging in our study is low (*n* = 12); moreover, they were particularly referred due to their high initial PSA level (Ø 393.9 ng/ml ± 903 vs. 5.7 ng/ml in Schmidkonz et al.). Therefore, no further conclusions can be drawn from this subgroup. But we hypothesize that [^99m^Tc]Tc-PSMA-I&S could be a valuable SPECT agent for detection of lymph node and bone metastasis in a significant proportion of the patients which has to be proven in further studies with histopathological validation.

### Restaging in advanced recurrent PCa

In advanced and symptomatic stages of PCa, therapeutic decisions are increasingly individual and highly depend on the patient’s preference, comorbidities, life expectancy, localization of metastases and general tumor load [12]. Therefore, unlike detection of metastatic disease in primary PCa and lesion detection of BCR, the use of imaging for restaging and general therapy monitoring is less standardized, and generally no recommendations exist. Also, to the best of our knowledge, no systematic studies on the superiority of either imaging modality in restaging and therapy monitoring of advanced PCa exist. Overall, PSMA has been shown to be upregulated on de-differentiated PCa, whereas the PSA level may decrease with increasing neoplastic de-differentiation [13]. Therefore, PSMA-targeted imaging may be increasingly used as a marker of therapy response [14].

In our analysis, patients with advanced disease were referred mainly for monitoring of antiandrogen therapy, chemotherapy, radiation therapy, and ^177^Lu-PSMA therapy. 21.9% of the scans (*n* = 46) were requested for these indications. At a median PSA level of 101.3 ng/ml, PSMA-positive tumor tissue could be detected in 84.8% of the scans. From our data no conclusions can be drawn on the diagnostic performance of [^99m^Tc]Tc-PSMA-I&S-SPECT/CT in comparison to other imaging modalities that are routinely recommended in advanced prostate cancer (e.g., CT, bone scintigraphy, whole body MRI). Also, no assumptions on a potential change in therapeutic management can be made from our study. However, based on the number of referrals and the spectrum of clinical questions by the referrals one may assume that there is a role for PSMA-targeted SPECT-imaging in the future to get a comprehensive overview on the whole body tumor load or on focal metastatic lesions that underwent targeted therapy.

#### Limitations

Our single-center retrospective study has several limitations. In most of the patients [^99m^Tc]Tc-PSMA-I&S-SPECT/CT was performed as an alternative imaging modality due to the unavailability of timely PSMA-PET/CT. Due to the lack of details in clinical information (e.g., prior therapies, conventional imaging results), no assessment of the performance of [^99m^Tc]Tc-PSMA-I&S-SPECT/CT in direct comparison to other conventional imaging modalities was possible. PMSA-positivity of tumor tissue was only assessed by visual means, and no quantitative analysis of PSMA expression was performed. Histopathology was not available for lesions detected on [^99m^Tc]Tc-PSMA-I&S-SPECT/CT, thus sensitivity and specificity could not be calculated.

## Conclusions

In this study, we report on the first diagnostic use of [^99m^Tc]Tc-PSMA-I&S-SPECT/CT in PCa. Despite the limitations of this retrospective single-center study our results indicate that [^99m^Tc]Tc-PSMA-I&S-SPECT/CT can be widely applied for diagnostic indications like BCR, primary staging, restaging in advanced PCa. Although a significant degree of hepatic, gastrointestinal, and urinary tracer excretion at the selected imaging time point (5 h post injection) was observed, low-dose CT usually allowed for good discrimination between tumor and non-tumor findings. At low PSA levels (< 4 ng/ml), detection rates of [^99m^Tc]Tc-PSMA-I&S-SPECT/CT were limited and should thus only be considered for lesion detection in BCR if imaging with PET or alternative high-affinity SPECT agents are unavailable. At higher PSA levels (> 4 ng/ml), [^99m^Tc]Tc-PSMA-I&S-SPECT/CT provided high detection rates in BCR. [^99m^Tc]Tc-PSMA-I&S-SPECT/CT was also suitable for primary staging and for restaging of advanced recurrent PCa. Our results are promising and warrant more systematic analyses and prospective clinical trials to further assess diagnostic performance of [^99m^Tc]Tc-PSMA-SPECT/CT in PCa.

## Data Availability

The datasets used and/or analyzed during the current study are available from the corresponding author on reasonable request.

## Abbreviations

BCR: Biochemical recurrence
CT: Computed tomography
mCRPC: Metastatic castration resistant prostate cancer
MRI: Magnetic resonance imaging
PCa: Prostate cancer
PET: Positron emission tomography
PSA: Prostate specific antigen
PSMA: Prostate specific membrane antigen
SPECT: Single photon emission computed tomography
